# Supplementary material for: Effect of Lauric vs. Oleic Acid-Enriched Diets on Leptin Autoparacrine Signalling in Male Mice
Source: Biomedicines. 2022 Aug 2;10(8):1864. doi: 10.3390/biomedicines10081864 (PMC9405789; doi:10.3390/biomedicines10081864)
Supplement: Supplementary file 1 [file biomedicines-10-01864-s001.zip › biomedicines-1816312-supplementary.pdf]

Effect of lauric *vs* oleic acid-enriched diets on leptin autoparacrine signalling in male mice

Jesús Fernández-Felipe[1+], Adrián Plaza[1,2+], Gema Domínguez[3], Javier Pérez-Castells[3], Victoria Cano[1], Francesco Cioni[1], Nuria Del Olmo[4], Mariano Ruiz-Gayo[1], Beatriz Merino[1]

SUPPLEMENTARY MATERIAL

Table S1. Diet composition. Oleic, palmitic and lauric acid content was quantified by liquid chromatography from saponified samples of high-oleic sunflower oil and palm kernel oil used for diets' manufacturing.

	SD	UOLF	SOLF
Energy density (kcal/g)	3.1	5.3	5.3
Energy from proteins (%)	27.2	9.9	9.8
Energy from carbohydrates (%)	55.5	20.5	20.4
Energy from lipids (%)	17.5	69.6	69.8
Total proteins (%)	21.0	13.1	12.9
Total carbohydrates (%)	42.9	27.1	26.9
Total lipids (%)	6.0	40.8	41.2
Oleic acid (%)	1.2	31.3	10.0
Palmitic acid (%)	0.7	3.0	6.1
Lauric acid (%)	-	-	19.8
Other fatty acids (%)	4.17	6.13	5.21

Table S2. Bonferroni adjusted P values corresponding to the effect of dietary treatment and acute leptin administration on relative phosphorylation levels of Tyr^{705}-STAT3, Ser^{473}-Akt and Tyr^{172}-AMPK in Sc-WAT.

**Diet**	**SD**	**SOLF**		**UOLF**	
	**Leptin**	**Saline**	**Leptin**	**Saline**	**Leptin**
**pSTAT3/STAT3**	*vs* SD+saline P<0.01	*vs* SD+saline P<0.05	*vs* SOLF+saline ns	*vs* SD+saline ns	*vs* UOLF+saline P<0.01
**pAkt/Akt**	*vs* SD+saline P<0.01	*vs* SD+saline P<0.05	*vs* SOLF+saline ns	*vs* SD+saline ns	*vs* UOLF+saline P<0.05
**pAMPK/AMPK**	*vs* SD+saline P<0.01	*vs* SD+saline P<0.05	*vs* SOLF+saline ns	*vs* SD+saline ns	*vs* UOLF+saline P<0.05

Table S3. Bonferroni adjusted P values corresponding to the effect of dietary treatment and acute leptin administration on relative phosphorylation levels of Tyr[705]-STAT3, Ser[473]-Akt and Tyr[172]-AMPK in Vis-WAT.

Diet	SD	SOLF		UOLF	
	**Leptin**	**Saline**	**Leptin**	**Saline**	**Leptin**
**pSTAT3/STAT3**	*vs* SD+saline<br>$P<0.05$	*vs* SD+saline<br>ns	*vs* SOLF+saline<br>ns	*vs* SD+saline<br>ns	*vs* UOLF+saline<br>$P<0.001$
**pAkt/Akt**	*vs* SD+saline<br>$P<0.01$	*vs* SD+saline<br>$P<0.05$	*vs* SOLF+saline<br>ns	*vs* SD+saline<br>ns	*vs* UOLF+saline<br>$P<0.05$
